# Comparative Transcriptome Analysis between Two Potato Cultivars in Tuber Induction to Reveal Associated Genes with Anthocyanin Accumulation

**DOI:** 10.3390/ijms23073681

**Published:** 2022-03-27

**Authors:** Ju Young Ahn, Jaewook Kim, Ju Yeon Yang, Hyun Ju Lee, Soyun Kim, Kwang-Soo Cho, Sang-Ho Lee, Jin-Hyun Kim, Tae-Ho Lee, Yoonkang Hur, Donghwan Shim

**Affiliations:** 1 Department of Biological Sciences, Chungnam National University, Daejeon 34134, Korea; wnduds357@naver.com (J.Y.A.); jkim6403@cnu.ac.kr (J.K.); wjs508@hotmail.com (J.Y.Y.); ju0934@hanmail.net (H.J.L.); soyun9096@naver.com (S.K.); 2 Highland Agriculture Research Institute, National Institute of Crop Science, Rural Development Admin-istration, Pyeongchang 25342, Korea; kscholove@korea.kr; 3 Department of Biomedical Engineering, Mokwon University, Daejeon 35349, Korea; ish1004@mokwon.ac.kr; 4 Division of Genomics, National Institute of Agricultural Sciences, Jeonju 54874, Korea; kimzz14@korea.kr (J.-H.K.); thlee0@korea.kr (T.-H.L.)

**Keywords:** Jayoung, tuber induction, flesh color, anthocyanin accumulation, transcriptome

## Abstract

Anthocyanins are generally accumulated within a few layers, including the epidermal cells of leaves and stems in plants. *Solanum tuberosum* cv. ‘Jayoung’ (hereafter, JY) is known to accumulate anthocyanin both in inner tissues and skins. We discovered that anthocyanin accumulation in the inner tissues of JY was almost diminished (more than 95% was decreased) in tuber induction condition. To investigate the transcriptomic mechanism of anthocyanin accumulation in JY flesh, which can be modulated by growth condition, we performed mRNA sequencing with white-colored flesh tissue of *Solanum tuberosum* cv. ‘Atlantic’ (hereafter, ‘Daeseo’, DS) grown under canonical growth conditions, a JY flesh sample grown under canonical growth conditions, and a JY flesh sample grown under tuber induction conditions. We could identify 36 common DEGs (differentially expressed genes) in JY flesh from canonical growth conditions that showed JY-specifically increased or decreased expression level. These genes were enriched with flavonoid biosynthetic process terms in GO analysis, as well as gene set enrichment analysis (GSEA) analysis. Further in silico analysis on expression levels of anthocyanin biosynthetic genes including rate-limiting genes such as *StCHS* and *StCHI* followed by RT-PCR and qRT-PCR analysis showed a strong positive correlation with the observed phenotypes. Finally, we identified StWRKY44 from 36 common DEGs as a possible regulator of anthocyanin accumulation, which was further supported by network analysis. In conclusion, we identified StWRKY44 as a putative regulator of tuber-induction-dependent anthocyanin accumulation.

## 1. Introduction

Anthocyanins, water-soluble vacuolar pigments accumulated by the addition of sugars to anthocyanidins that are biosynthesized through the flavonoid pathway [1], contribute to plant color variations in flowers, leaves, stems, roots, and fruits. The anthocyanins attract pollinators and seed distributors [2,3,4], and protect plants from abiotic stresses, such as ultraviolet (UV) radiation, cold, drought and nutrition depletion, and biotic stresses, such as microbial infection [5,6,7,8,9,10,11,12]. Since anthocyanins exhibit potent antioxidant activity [13,14,15], they are expected to promote human health by lowering the incidence of cardiovascular diseases and chronic and degenerative diseases [16,17,18]. Potato (*Solanum tuberosum* L.) is the fourth largest crop in terms of production and the world’s most important non-grain food crop. Colored potatoes have potential nutritional benefits compared with colorless potatoes [19], therefore, colored phenotype has been targeted for breeding.

The anthocyanin content in plants depends on the balance between biosynthesis and degradation. Anthocyanin metabolism is regulated through well-known pathways triggered by developmental and environmental cues [20]. Anthocyanin biosynthesis in plants is controlled by several genes, encoding structural proteins for the flavonoid synthetic pathway and relevant transcription factors. Initially, the early flavonoid reactions catalyzed by early biosynthetic genes (EBGs) included chalcone synthase (*CHS*), chalcone isomerase (*CHI*), and flavonol 3-hydroxylase (*F3H*). Three redundant R2R3 MYB transcription factors (TFs), MYB11, MYB12, and MYB111, were shown to regulate EBGs [21]. Late biosynthetic genes (LBGs) consist of dihydroflavonol-4-reductase (*DFR*), leucoanthocyanidin dioxygenase (*LDOX*/anthocyanidin synthase *ANS*), and UDP-glucose:flavonoid-3-O-glycosyl-transferase (*UF3GT*). These LBGs were shown to be regulated by the ternary MYB-bHLH-WD40 (MBW) protein complex, which is composed of R2R3-MYB, basic helix–loop–helix (bHLH), and WD40-repeat proteins [1,22]. In Arabidopsis, other R2R3-MYB transcription factors such as PRODUCTION OF ANTHOCYANIN PIGMENTATION 1 (PAP1)/MYB75, PAP2/MYB90, MYB113, and MYB114 were identified to regulate LBGs [23,24]. The bHLH transcription factors TRANSPARENT TESTA 8 (TT8) and ENHANCER OF GLABRA 3 (EGL3), and one WD40-repeat protein, TRANSPARENT TESTA GLABRA 1 (TTG1), have been identified [25,26,27,28]. The formation of the MBW complex regulates the spatiotemporal expression pattern of LBGs in response to developmental and environmental factors [12,24,28,29].

Over 600 anthocyanins have been identified in nature [30]. In plants, the most common anthocyanins are the derivatives of six widespread anthocyanidins, namely pelargonidin, cyanidin, delphinidin, peonidin, petunidin, and malvidin [31]. The most abundant anthocyanins in purple-colored *S. tuberosum* cv. ‘Jayoung’ (hereafter, JY) are peonidin, petunidin, delphinidin, and malvidin [32]. To identify genes critical for anthocyanin accumulation in potatoes, transcriptomic analysis with different colored potatoes has been carried out [32,33,34,35,36]. These studies identified EBGs, LBGs, and anthocyanin accumulation regulatory genes, including regulators identified from other plants, and demonstrated that novel regulators are highly associated with color phenotypes in potatoes. For example, StAN1 (potato ANTHOCYANIN1) was identified as novel regulator of anthocyanin biosynthesis in potato tubers and leaves [37,38].

JY is a pigmented potato cultivar originated from a cross between the white-colored Atlantic and deep dark-purple-colored ‘AG34314′ cultivars through the potato breeding program of the National Institute of Highland Agriculture Research Center in 2003 [39]. We previously performed mRNA-seq analysis with sprout tissues from JY and identified several genes that were positively correlated with JY pigmentation [32]. Meanwhile, we discovered that the flesh tissue of JY did not accumulate with anthocyanin under tuber induction conditions. This phenotype triggered us to hypothesize that common anthocyanin accumulation mechanisms which can explain both tissue culture phenotype and cultivar difference in flesh tissue might exist. To figure out common transcriptomic mechanism as in our hypothesis, we performed mRNA-seq analysis with a flesh tissue sample of *S.tuberosum* cv ‘Atlantic’ (hereafter, ‘Daeseo’, DS) and JY in nutrient growth conditions, and with a JY flesh sample in tuber induction conditions. Anthocyanin-related gene expression was positively correlated with a reduced anthocyanin accumulation phenotype including biosynthetic genes, as expected. Moreover, we could identify one possible regulator of JY-specific anthocyanin accumulation, StWRKY44, from differentially expressed genes. Network analysis not only confirmed our suggestion but also supported the potential regulator StTT8, identified in previous work.

## 2. Results

### 2.1. Tuber Growth Induction Introduced a Tuber with Pale-Colored Flesh in JY

**Figure 1:**
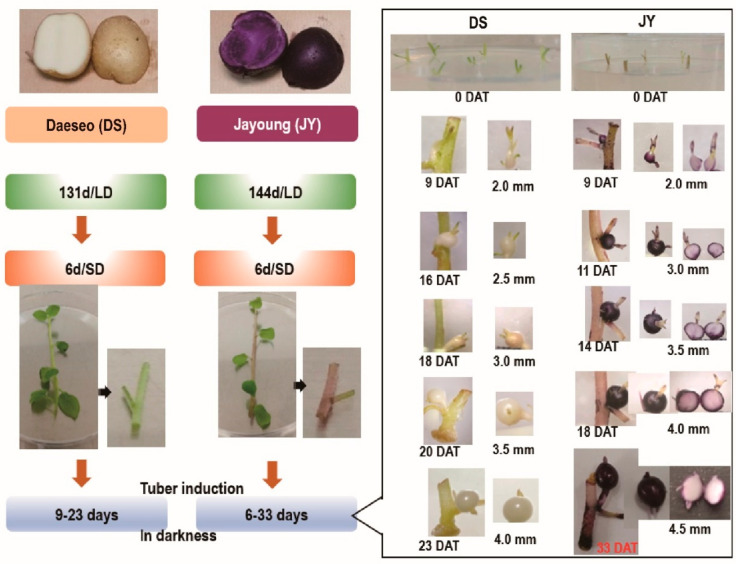
Tuber induction (TI) process from single-node cuttings and phenotypes of each stage. DS (‘Daeseo’) and JY (‘Jayoung’) tissue cultures were kept for 131 days and 144 days, respectively, under long-day conditions (16 h light /8 h dark) in a culture room set to 22 ± 0.5 °C and 110 µmol m^−2^ s^−1^ light intensity. Then, plants were transferred to short-day (SD) conditions (8 h light /16 h dark) for 6 days before preparing a single node. Tuber induction was performed in darkness for the indicated days. Tuber diameters are expressed as mm, and JY tubers were cross-sectioned to show anthocyanin accumulation. DAT indicates day after transfer to darkness. Single-node cuttings on induction medium are shown at 0 DAT.

**Figure 2:**
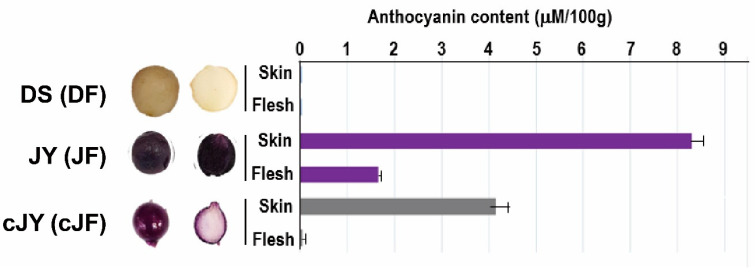
Tuber morphology and anthocyanin content assay indicates cJF had lower anthocyanin contents as DF. DS (DF in mRNAseq), JY (JF in mRNAseq), and cJY (cJF in mRNAseq). cJY, cultured ‘Jayoung’; DF, ‘Daeseo’ flesh; JF, ‘Jayoung’ flesh; cJF; cultured ‘Jayoung’ flesh.

Tuber growth was induced with stem samples of long-day (LD)-grown potato plants transferred to short-day (SD) conditions for 6 days in the dark for 6 to 33 days (Figure 1). DS required a shorter growth period than JY in order to induce tuber growth. In nutrient culture conditions, JY accumulated anthocyanin in every tissue at any phase of growth, with minor variations. On the other hand, DS accumulated significantly less anthocyanin than JY in the same conditions (Figure S1). Moreover, the flesh tissue of JY in tuber induction conditions showed significantly less accumulation of anthocyanin compared with the flesh tissue of JY grown in nutrient culture conditions (Figure 1). Similar to a previous study on other purple-colored cultivars, the skin tissue of JY was measured to have higher anthocyanin contents than in flesh in canonical growth conditions. Interestingly, the flesh tissue of JY in tuber induction conditions had anthocyanin contents as low as in DS samples (Figure 2).

### 2.2. Functional Annotation Reveals Anthocyanin Precursor Biosynthetic Genes Are Up-Regulated in Nutrient-Cultured JY Flesh Tissue

**Figure 3:**
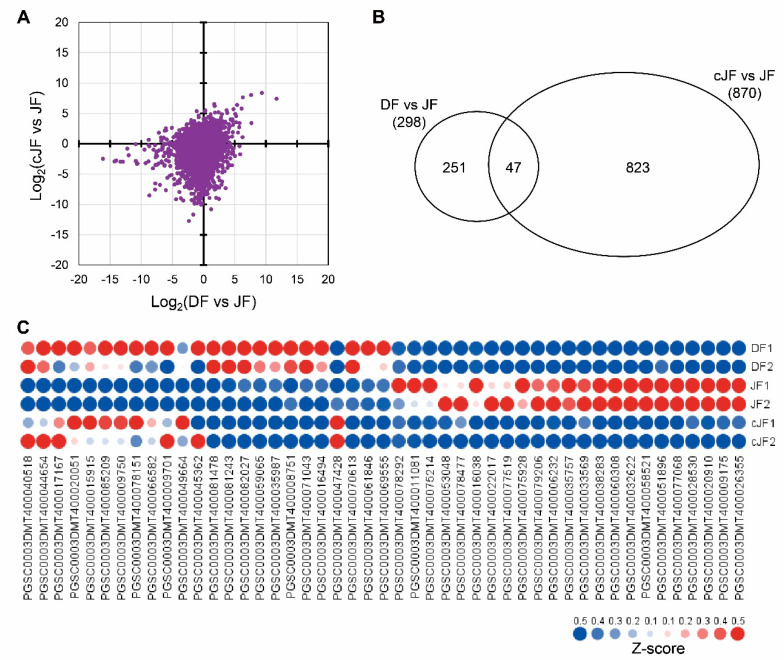
DEG (differentially expressed genes) analysis of DF, JF, and cJF identified 47 genes. (**A**) Expression patterns in dot plot. The x axis indicates log_2_ value of TPM (transcripts per million) in DF divided by TPM in JF for each gene, and the y axis indicates log_2_ value of TPM in cJF divided by TPM in JF for each gene. (**B**) Venn diagram depicting common and specific DEGs. (**C**) Heatmap of common DEGs. Z-score for each gene was used. Numbers in sample names indicate biological replicate number.

To figure out how the flesh tissue of JY grown in tuber induction conditions does not accumulate anthocyanin as in DS, we performed mRNA sequencing to analyze the genome-wide transcriptome of flesh tissues from nutrient-cultured DS (hereafter, DF), nutrient-cultured JY (hereafter, JF), and JY grown in tuber induction conditions (hereafter, cJF). We compared the TMM (trimmed mean of M-values) and normalized TPM (transcript per million) values of JF with DF or cJF, with JF being the only anthocyanin-accumulated sample. Then, we examined how the total transcriptome was changed in JF compared with DF or cJF. The correlation efficient of DF vs. JF and cJF vs. JF was 0.266, which indicates that no significant correlation was found (Figure 3A). Thus, we analyzed differentially expressed genes (DEG) comparing DF vs. JF and cJF vs. JF with the criteria of 4-fold change and FDR (false discovery rate, adjusted *p*-value) < 0.05. A total of 298 genes were identified as DEG in DF vs. JF, while 870 genes were identified in cJF vs. JF. Among these, 47 genes were identified as common DEG between two groups, which was a significantly greater number of genes than expected, with a hypergeometric p-value of 2 × 10^−21^ (Figure 3B). Among 47 genes, 23 genes were up-regulated and 13 genes showed decreased expression level (Figure 3C; Table S2).

**Figure 4:**
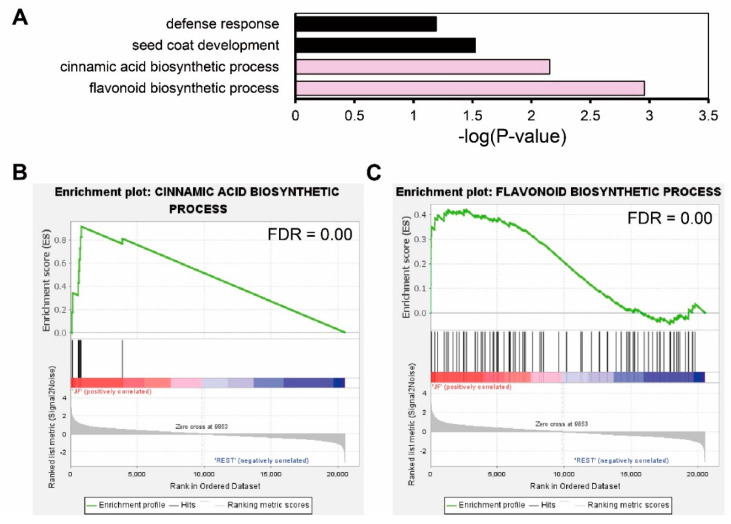
Gene Ontology analysis of common DEGs regulated in same direction indicates flavonoid biosynthesis was up-regulated in JF. (**A**) GO analysis of common DEGs. Significance of enrichment is depicted by -log(*p*-value) in the x axis. Pink filled bars indicate anthocyanin precursor biosynthetic process terms. (**B**,**C**) GSEA (gene set enrichment analysis) of flavonoid biosynthetic process genes. GSEA rank was calculated by JF vs. REST (DF and cJF). Common DEGs that were regulated in same direction both in JF vs. DF and JF vs. cJF were used as a query.

Using 36 JF-specific DEGs, we performed functional annotation with DAVID GO analysis. Four GO terms in biological processes were enriched: defense response (*p*-value = 0.0015), seed coat development (*p*-value = 0.00047), cinnamic acid biosynthetic process (*p*-value = 0.00002), and flavonoid biosynthetic process (*p*-value = 0.000055). Though we could not detect the direct enrichment of anthocyanin-related GO terms, the flavonoid biosynthetic process and cinnamic acid biosynthetic process which are precursor biosynthetic processes of anthocyanin, were enriched (Figure 4A). We further verified the enrichment of these GO terms by gene set enrichment analysis (GSEA) in the whole transcriptome, comparing JF with DF or cJF. Genes annotated in the cinnamic acid biosynthetic process (FDR = 0.00) and flavonoid biosynthetic process (FDR = 0.00) were significantly enriched and JF-specifically up-regulated (Figure 4B,C), as well as seed coat development genes (FDR = 0.00), but not defense-response-related genes (FDR = 0.07) (Figure S2). These data indicate flavonoid biosynthetic or cinnamic acid biosynthetic genes are JF-specifically up-regulated, which positively correlates with JF-specific anthocyanin accumulation.

### 2.3. In Silico Analysis and qRT-PCR Confirms Anthocyanin Biosynthetic Genes Are Up-Regulated in JF

We could also identify the JF-specific up-regulated gene cluster (Cluster10) and down-regulated gene cluster (Cluster12) by cluster analysis (Figure S3A). A total of 13 genes were included in Cluster10, while 2 genes were included in Cluster12. Of 13 genes in Cluster10, 8 genes known to positively regulate anthocyanin contents or anthocyanin biosynthetic genes were found, and 1 gene was found to be an anthocyanin biosynthetic gene in Cluster12 (Figure S3B).

**Figure 5:**
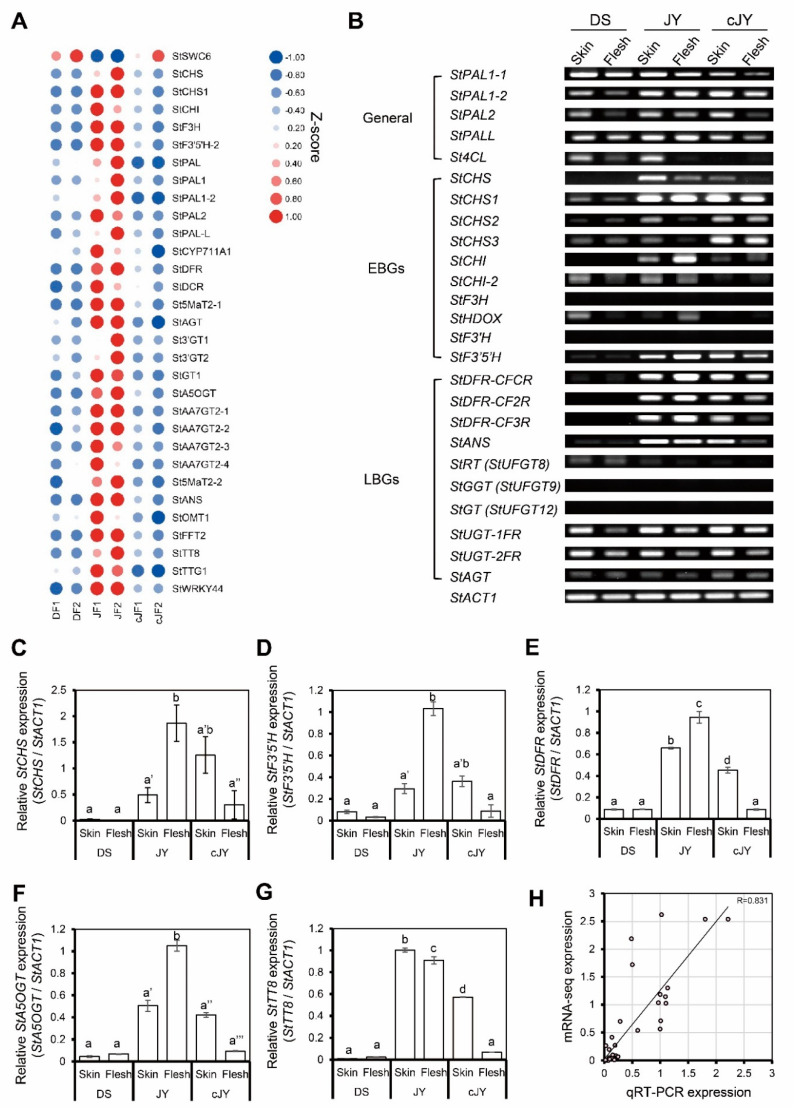
Anthocyanin-related genes were positively correlated with JF. (**A**) Heatmap of anthocyanin-related genes from mRNA-seq. Z-scores for each gene were used. Numbers in sample names indicate biological replicate number. (**B**) RT-PCR of anthocyanin biosynthetic genes. General indicates general pathway genes in flavonoid biosynthetic pathway, EBG indicates early biosynthetic genes, and LBG indicates late biosynthetic genes. *StACT1* was used as an internal control. (**C**–**G**) qRT-PCR of anthocyanin-related genes. Relative expression levels of each gene were determined by 2^−ΔΔCT^ method. Letters represent significant differences based on one-way ANOVA and Tukey’s test (*p* < 0.05). Two biological replicates were analyzed. (**C**) Relative expression level of *StCHS*. (**D**) Relative expression level of *StF3′5′H*. (**E**) Relative expression level of *StDFR*. (**F**) Relative expression level of *StA5OGT*. (**G**) Relative expression level of *StTT8*. (**H**) Linear regression analysis between qRT-PCR expression and mRNA-seq data. Relative expression levels of each gene were determined by 2^−ΔΔCT^ method, as indicated in the x axis, while the TMM-normalized TPM of target genes divided by that of *StACT1* is indicated in the y axis.

We further analyzed anthocyanin-related gene expression patterns in our transcriptome data and found 32 genes to be positively correlated with the JF-specific anthocyanin phenotype, while 27 genes were anthocyanin biosynthetic genes (Figure 5A). To validate these in silico data, we performed reverse-transcript PCR (RT-PCR) analysis on anthocyanin biosynthetic genes. Expression levels of many biosynthetic genes (11 out of 22 genes tested, including *StPAL*, *StCHS,* and *StCHI*) were decreased in the flesh tissue of JY in tuber induction (hereafter cJY) compared with JY samples. Interestingly, anthocyanin biosynthetic genes of general steps, including *PAL*s (*phenyl alanine ammonia-lyase*), were expressed at a comparable level with JY samples in DS, but early biosynthetic genes (EBGs) and many late biosynthetic genes (LBGs) such as *StCHS* and *StDFR* were down-regulated in DS compared with JY (Figure 5B). We performed quantitative real-time PCR (qRT-PCR) on several biosynthetic genes and previously suggested regulator genes. Biosynthetic genes had the highest expression in nutrient-cultured JY flesh tissue, and skin tissues showed a comparable level of biosynthetic genes in both JY and cJY. The flesh tissue of cJY showed a similar expression level to DS samples in our experiment (Figure 5C–F). One anthocyanin biosynthesis positive regulator, *TESTA TRANSPARENT 8* (*TT8*), was highly expressed in JY skin and flesh and cJY skin but was relatively less expressed in DS skin, flesh, and cJY flesh tissues (Figure 5G), which is consistent with our previous results [32]. Linear regression analysis of the qRT-PCR experiment and mRNA-seq experiment showed a correlation efficient of 0.831, which is a highly positive correlation (Figure 5H). Thus, our transcriptome data and qRT-PCR results suggest that the flesh tissue of cJY grown in tuber induction media showed a lower expression of anthocyanin biosynthetic genes such as DS, which was positively correlated with phenotype.

### 2.4. WRKY44 Might Be a Central Regulator of Anthocyanin Accumulation

**Figure 6:**
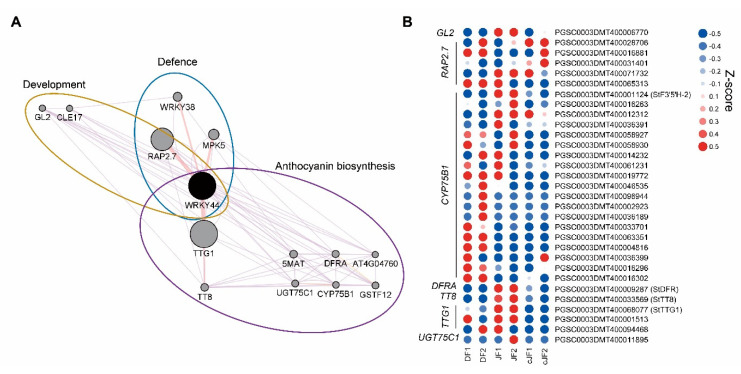
Network analysis of WRKY44 and expression patterns of WRKY44 network genes. (**A**) Network analysis of WRKY44. Pale purple lines indicate co-expression network and pale red lines indicate physical interaction. (**B**) Heatmap of WRKY44 network genes. Z-scores for each gene were used. Numbers in sample names indicate biological replicate number.

DEG analysis indicated that a portion of transcription factors regulating anthocyanin accumulation were JY-specifically up-regulated, such as MYB domain-containing protein 3 (MYB3), transcription factor TT8 (TT8), and WRKY transcription factor 44 (WRKY44), which were positively correlated with our phenotype (Figure 3C; Table S2). To identify the putative regulator of anthocyanin accumulation in the flesh tissue of JY, we performed network analysis and found WRKY44 as a putative central regulator of our phenotype (Figure 6A). Moreover, WRKY44 regulated many anthocyanin biosynthesis-related genes as a putative complex with TRANSPARENT TESTA GLABRA 1 (TTG1) and TT8. Anthocyanin biosynthetic genes in the WRKY44 network were more highly expressed in JF than in DF or cJF, such as *flavonoid-3′,5′-hydrolase 2* (*St3′5′H-2*) and *dihydroflavonol 4-reductase A* (*StDFRA*), as well as *TTG1* and *TT8,* which were known to positively regulate anthocyanin accumulation (Figure 6B). Taken together, our results suggest a novel WRKY44-bHLH-WD40 module in the regulation of anthocyanin biosynthesis.

## 3. Discussion

### 3.1. Tuber Induction Condition Suppresses Flavonoid Biosynthetic Pathway Genes

Our tuber induction condition provides information on the regulation of anthocyanin accumulation in potato flesh tissue (Figure 2 and Figure 3) by both difference of cultivar and growth condition difference. Though DS and JY is different cultivar, JY originated by crossing natural deep-purple cultivar ‘AG34314’ and DS which makes our sample set of transcriptomic analysis to be appropriate samples to identify transcriptomic mechanism of anthocyanin accumulation in potato [39]. A series of in silico analyses revealed that the expression levels of flavonoid biosynthetic pathway genes were suppressed in cJF and in DF compared with JF. This in silico observation was confirmed by qRT-PCR, in a tissue-specific manner (Figure 4 and Figure 5). CHS and CHI catalyze 4-coumaroyl-CoA into naringenin; thus, these are considered to be rate-limiting enzymes in flavonoid biosynthetic pathway [40,41]. Our data indicate that both genes are up-regulated in anthocyanin-accumulating tissues (Figure 5), which provides convincing evidence on the observed phenotype. We observed that the cJY tuber was even more colorless than DS flesh tissue, which was unexpected (Figure 2). A possible explanation for this phenotype can also be found in our transcriptome, in the expression level of *PGSC0003DMT400061846* (*StPSY1*, phytoene synthase 1). PSY is known to be a rate-limiting step of carotenoid biosynthesis, which catalyzes carotene as the first step in a two-step reaction [42]. *StPSY1* had the highest expression level in DF, and the lowest expression level in cJF. Taken together, we identified anthocyanin as a dominant pigment than carotenoid in JY pigmentation due to their over-accumulation.

### 3.2. Possible Enzymes of JY-Specific Purple Color

Early steps of anthocyanin biosynthesis include the flavonoid biosynthetic pathway and anthocyanin biosynthetic pathway, which generate red, blue, or purple pigments. An interesting observation from our data is that expression level of many of the flavonoid biosynthetic pathway genes and anthocyanin biosynthetic pathway genes are significantly decreased in DS regardless of tissue (Figure 5). This observation enables us to suspect cultivar-specific regulators of anthocyanin accumulation. Four transcription factors which were known to regulate anthocyanin accumulation were identified as DEGs in comparison with DF and JF, *PGSC0003DMT400078477* (*MYB3*), *PGSC0003DMT400036281* (*MYB6*), *PGSC0003DMT400028530* (*WRKY44*), and *PGSC0003DMT400036284* (*MYB90*, *PAP2*) (Table S3). MYB3 is a member of the R2R3 MYB transcription factor family known to regulate anthocyanin accumulation; however, there is evidence to claim that it both induces and inhibits anthocyanin accumulation [43,44]. MYB6 is also one of the R2R3 MYB transcription factors known to regulate anthocyanin accumulation; controversial evidence also exists regarding anthocyanin regulation [45,46]. WRKY44 was also found to be a DEG in comparison with cJF and JF. MYB90 is also referred to as PAP2 (production of anthocyanin pigment 2) and positively regulates anthocyanin accumulation [47], which was up-regulated in DS compared with JY. Thus, at least in DEG analysis, we could not identify a DS cultivar-specific regulator of anthocyanin biosynthesis.

JY is more highly accumulated with cyanidin derivatives and delphinidin/petunidin/malvidin derivatives than DS, which are known to be red/purple pigments, respectively [26]. Anthocyanin acyltransferase (AAT), which catalyzes the transferring reaction of acyl groups of anthocyanin derivatives, and a portion of glucosyltransferase, which transfers the glucosyl group of anthocyanin derivatives, are the main enzymes in terms of variable pigmentation. A putative *StAAT* (*PGSC0003DMT400041528*) and four *StGT* (glucosyl transferase, *PGSC0003DMT400035859*, *PGSC0003DMT400058739*, *PGSC0003DMT400021173*, and *PGSC0003DMT400030871*) were JY-specifically up-regulated (Table S3). Though specific functions remain to be studied, these five genes might be responsible for the JY-specific accumulation of red- or purple-colored anthocyanin derivatives.

### 3.3. WRKY44 Might Be a Central Regulator of Anthocyanin Accumulation

DEG analysis indicated several transcription factors to be responsible for tuber induction phenotype, such as MYB3, TT8, and WRKY44 (Figure 3C; Table S2). Controversial results exist regarding the function of MYB3. TT8 is known to regulate the LBGs of anthocyanin in a positive manner [35]. Interestingly, WRKY44 was also known to up-regulate anthocyanin accumulation [48]. TT8 was known to require a second transcription factor to induce anthocyanin accumulation, and so we focused on WRKY44 (Figure 6). Moreover, WRKY44 interacts with TT8 and TTG1, which supports possible TT8 functionality in our transcriptome data (Figure 6). Our RT-PCR and qRT-PCR analyses on anthocyanin biosynthetic genes further imply putative WRKY44-TTG1-TT8 regulation (Figure 5).

WRKY44 is a putative causative transcription factor when we assume that the lower anthocyanin accumulation of JY flesh in tuber induction conditions has an identical mechanism to that of DS flesh (Figure 1 and Figure 2). However, it is possible that the lower anthocyanin accumulation of JY flesh in tuber induction conditions has a specific mechanism itself (cJF-specific phenotype hypothesis). Assuming this possibility, there were seven putative causative transcription factors which could be suggested by the transcriptome data; *PGSC0003DMT400051047* (*WRKY40*), *PGSC0003DMT400014949* (*WRKY53_1*), *PGSC0003DMT400031695* (*WRKY53_1*), *PGSC0003DMT400031899* (*bHLH14*), *PGSC0003DMT400015017* (*MYC4*), *PGSC0003DMT400067321* (*MYB15*), and *PGSC0003DMT400011748* (*MYB112*). MdWRKY40 was shown to enhance anthocyanin accumulation, but evidence was restricted in MYB111 overexpression callus, and not in wild type [49]. WRKY53 was shown to suppress anthocyanin accumulation, which does not explain our phenotype [50]. AtbHLH14 was shown to enhance anthocyanin accumulation, but it was redundant with three other bHLH transcription factors [51]. AtMYC4 was shown to enhance anthocyanin accumulation; thus, it may be a candidate transcription factor in the cJF-specific phenotype hypothesis [52]. AtMYB112 was shown to enhance anthocyanin accumulation in high light conditions or salinity stress conditions, which does not align with our tuber induction condition [53]. LrMYB15 was shown to enhance anthocyanin accumulation, and thus could be another candidate transcription factor of our phenotype [54].

### 3.4. Many Anthocyanin Biosynthesis Pathway Genes Are Putative WRKY44/TT8 Target Genes

Throughout our analysis, we suggest WRKY44 might be regulator both in cultivar-dependent and tuber induction-dependent anthocyanin accumulation with TT8 and TTG1. Till now, there is no strict evidence in WRKY44 and TT8 binding targets while they were known to regulate anthocyanin biosynthesis genes. WRKY44 was shown to up-regulate *F3H* while TT8 was shown to up-regulate *DFR*. To figure out putative regulatory effect of WRKY44 and TT8, we performed motif analysis with flavonoid biosynthetic pathway genes and putative anthocyanin biosynthetic genes. TT8 belongs to bHLH superfamily, thus expected to target G-box (CACGTG) motif while WRKY44 is expected to target W-box (TTGACW(c/t)). Thus, we searched both motifs in anthocyanin biosynthetic pathway genes and identified 29 of 295 genes (9.83%) had both G-box and W-box motifs, being putative TT8 and WRKY44 co-target. While 201 of 295 genes (68.1%) had G-box or W-box motif which they are entitled as putative target genes of TT8 or WRKY44 (Table S4). Of flavonoid biosynthetic genes, we analyzed expression patterns and identified strong positive correlation with anthocyanin accumulation phenotype. Moreover, 7/17 genes (41.1%) of flavonoid biosynthetic genes had G-box, and 12/17 genes (70.6%) of genes had W-box which was even higher ratio than all of analyzed genes in Table S4. Especially, expression patterns of *F3H* and *DFRA* showed high correlation with our phenotype, suggesting high possibility of TT8 and WRKY44 regulation on our anthocyanin accumulation phenotype of JY (Figure S4). Moreover, *StWRKY44* and *StTT8*, *StTTG1,* and *StWD40* were up-regulated in purple colored tissues (Figure 5G and Figure S5). Taken together, our data indicates TT8 and WRKY44 to be putative central regulator of JY-specific anthocyanin accumulation with TTG1 or WD40.

### 3.5. HY5 As a Putative Causative Factor of Anthocyanin Accumulation Phenotype

Our tuber induction condition includes dark incubation for a long time, which is not a canonical tuber induction method (Figure 1). Some of the transcription factors in the light signaling pathway are known to regulate anthocyanin accumulation, such as PHYTOCHROME INTERACTING FACTORs (PIFs) and ELONGATED HYPOCOTYL 5 (HY5). PIFs are transcription factors promoting de-etiolation, and are thus known to suppress anthocyanin biosynthesis genes in the dark, while HY5 induces anthocyanin biosynthesis via inducing anthocyanin biosynthetic gene expression [55,56]. The experimental conditions to induce anthocyanin involve a light stimulus with an external sucrose supplement, which coincides with our tuber induction methods. Meanwhile, PIFs are readily degraded by light stimulus and HY5 is active in light conditions while inactive in dark, due to proteasome-dependent degradation [57,58,59]. Indeed, the tuber induction conditions reduced the anthocyanin accumulation of skin and flesh tissues of JY (Figure 2), not in flesh tissue specifically. Thus, HY5 maybe the reason for the lesser accumulation of anthocyanin in JY flesh under tuber induction conditions, which warrants further study.

## 4. Materials and Methods

### 4.1. Potato Tissue Culture

Tissue culture stocks for DS and JY were obtained from Highland Agriculture Research Institute (National Institute of Crop Science, Rural Development Administration, Pyeongchang 25342, Republic of Korea). The stocks were cultured in Murashige and Skook (MS, Duchefa, Haarlem, The Netherland) medium containing MES (0.5 g/L, Duchefa, Haarlem, The Netherland), 1% sucrose (Duchefa, Haarlem, The Netherland) and 0.8% (*w*/*v*) phyto-agar (Duchefa, Haarlem, The Netherland) in an environmental growth chamber maintained at 22 °C, 16 h light/8 h dark photoperiod and 140 μmol m^−2^ s^−1^ light intensity. Before tuber induction (TI), plants were transferred to short-day (SD) (8 h light/16 h dark) conditions for 5–7 days. Samples for nutrient culture (NC) of DS and JY were cultured in the culture room, which had 20 °C and 16 h light/8 h dark photoperiod and 140 μmol m^−2^ s^−1^ light intensity of the Highland Agriculture Research Institute. All chemicals used in culture were purchased from Duchefa Biochemie (Haarlem, The Netherlands).

### 4.2. Tuber Induction

Single-node cuttings were conducted following a previous report [60]. Plantlets were grown in culture tubes under long-day conditions, and then transferred to SD conditions for 6 days. Plants were then cut to one-node stem pieces ca. 2 cm in length containing a resting axillary bud and a fully expanded leaf. Per plant three stem pieces containing the uppermost fully expanded leaves were taken. The leaves were removed, leaving about 1 cm of petiole. Node samples were cultured on the tuber induction medium, which consists of MS medium with 1/10 of nitrate salts (169 mg/L NH_4_NO_3_ (Sigma-Aldrich, St. Louis., MO, USA), 190 mg/L KNO_3_ (Sigma-Aldrich, St. Louis., MO, USA)), MES (0.5 g/L), 8% sucrose, 5 μM 6-benzylaminopurine (Sigma-Aldrich, St. Louis., MO, USA), and 0.8% (*w*/*v*) phyto-agar. Cultures were kept in the dark at 22 °C (Figure 1).

### 4.3. Anthocyanin Content Measurement

Anthocyanin content was measured as in previous report with minor modification [61]. Briefly, fresh weight of each sample was measured and powdered in liquid nitrogen, and added with 1% (*v*/*v*) HCl-methanol. Then, samples were incubated in darkness at room temperature for 24 h with gentle shaking, which followed by centrifugation of 13,000× *g*. Supernatant liquid of each sample was collected and absorbance was measured at 530, 620, and 650 nm. Then, relative anthocyanin content was calculated as following: R = (A_530_ − A_620_) − 0.1 × (A_650_ − A_620_) where A_530_, A_620_, and A_650_ indicates absorbance value of supernatant solutions at 530, 620, and 650 nm and R being relative anthocyanin content, respectively. Then, total anthocyanin content was calculated on the basis of reference material, cyanidin-3-O-glucoside (Sigma-Aldrich, St. Louis., MO, USA) as following [62]: R × MW × DF × 1000 × E. MW indicates molecular weight of cyanidin-3-O-glucoside (449.2 g/mol), DF indicates dilution factor, and E indicates the cuvette optical path length (1 cm).

### 4.4. RNA Sequencing and Analysis

Flesh tissues were obtained from tubers (ca. 3~4 mm in diameter) of DF, JF, and cJF, and frozen in liquid nitrogen and kept at −70 °C until use. Total RNA was isolated from each potato sample by using Trizol reagent (Invitrogen, Calsbad, CA, USA) according to the manufacturer’s protocols. Two biologically independent replications were performed for each sample. Then, 20 μg of samples with an RNA integrity number (RIN) > 8 were used for library construction. Each paired-end cDNA library was prepared according to the TruSeq RNA Sample Preparation Guide (Illumina, San Diego, CA, USA) and then sequenced on the Illumina HiSeq X Ten. Paired-end reads were cleaned using prinseq-lite version (v0.20.4, US) with the following parameters: min_len 50; min_qual_score 5; min_qual_mean 20; derep 14; trim_qual_left 20; trim_qual_right 20. Clean paired-end reads of each sample were aligned to the potato reference sequence using Bowtie2 (v2.4.1, US). The RSEM 1.3.0 software (v1.3.0, US) was used to obtain read counts and TMM-normalized TPM (trimmed mean of M value-normalized transcripts per million) values for each transcript. EdgeR version 3.16.5 (v3.16.5, US) was used to calculate the negative binomial dispersion across conditions for differential gene expression analysis. Genes were determined to be significantly differentially expressed if they showed > 4-fold change in expression, with a false discovery rate (FDR)-adjusted *p* < 0.001. DEG (differentially expressed genes) analysis was performed according to ref. [63] using Ensembl potato database annotation [63,64,65,66].

### 4.5. Functional Annotation and Network Analysis

BLAST program (v2.12.0+, US) with an e-value threshold of 1E^−5^ against *Arabidopsis thaliana* protein database was used for functional annotation of differentially expressed genes. Gene ontology (GO) term enrichment analysis was performed using DAVID [67], and enriched GO terms were determined by Fisher’s exact test (*p* < 0.05). Enriched GO genes were further analyzed with gene set enrichment analysis (GSEA, v4.1.0, US), as in [68,69]. Network analysis was performed with GeneMANIA app (v3.5.2, US) in Cytoscape (v3.9.0, US) [70,71] with WRKY44 as query gene.

### 4.6. Expression Analysis of Potato Genes

Anthocyanin biosynthetic genes or flavonoid biosynthetic genes were selected to analyze gene expression on the basis of mRNA-seq data. Primer sequences were designed by using potato sequences in Spud DB (Potato Genomics Resource: http://solanaceae.plantbiology.msu.edu/, accessed on 20 March 2022). Total RNA was extracted from tuber tissues (skin and flesh) isolated from 3~4 mm diameter tubers with TRIzol Reagent (Thermo Scientific, Waltham, MA, USA), and 1 μg of total RNA was treated with RQ1 RNase-free DNase (Promaga, Madison, WI, USA) at 65 °C for 10 min. First-strand cDNA was synthesized using oligo dT primers and Revertra Ace Kit (TOYOBO, Osaka, Japan). Primers used in this study are listed in Table S1. Quantitative real-time PCR (qRT-PCR) was performed on the MiniOpticon system (Bio-Rad, Hercules, CA, USA) using SYBR Green Realtime Master Mix (TOYOBO, Osaka, Japan). PCR conditions were as follows: denaturation at 95 °C for 10 min, followed by 40 cycles of 94 °C for 30 s, 58 °C for 30 s, and 72 °C for 30 s. Fluorescence values were measured at the last step of each cycle. All analyses were performed with three biological replicates. Transcript levels of target genes were normalized relative to the Actin1, and analyzed using the 2^−ΔΔCT^ method [72]. Semi-quantitative RT-PCR was performed as follows: initial denaturation at 94 °C for 5 min, followed by 27 cycles of 94 °C for 30 s, 60 °C for 30 s, and 72 °C for 30 s, and a final extension at 72 °C for 7 min. DNA fragments amplified by semi-quantitative RT-PCR were separated on 1.5% agarose (Thermo Scientific, Waltham, MA, USA) gels and stained with ethidium bromide (Thermo Scientific, Waltham, MA, USA).

## 5. Conclusions

Tuber induction of purple-colored *S.tuberosum* cv. ‘Jayoung’ inhibits anthocyanin accumulation, which was more dramatic in flesh tissue. To figure out flesh-specific anthocyanin accumulation in the transcriptome, we performed mRNA-sequencing analysis with flesh tissues of *S.tuberosum* cv. ‘Daeseo’ (‘Atlantic’) and JY grown in canonical conditions, along with that of JY grown in tuber induction conditions. Our in silico analysis and a series of molecular analyses revealed that *S.tuberosum* cv. ‘Daeseo’ and the flesh tissue of JY grown in tuber induction conditions were down-regulated with anthocyanin biosynthetic genes, including the rate-limiting enzyme of flavonoid biosynthesis, *StCHS*. Moreover, we identified that StWRKY44 might modulate anthocyanin biosynthesis both in a cultivar-specific and growth-condition-specific manner through network analysis. Our analysis provides possible transcriptomic mechanism of anthocyanin accumulation which can explain cultivar difference and physiological response. Thus, our result not only provides the putative transcription factors that might be causative of such phenomena in cultivar difference alongside with previous works, but also provides single network being associated with anthocyanin accumulation in both cultivar difference and physiological response.

## Data Availability

The raw data were deposited in the Korean Nucleotide Archive (KoNA, https://kobic.re.kr/kona, accessed on 20 March 2022) with the accession ID PRJKA210107 and NCBI’s Sequence Read Archive (SRA) with the accession ID PRJNA818084.

## Supplementary Materials

The following supporting information can be downloaded at: https://www.mdpi.com/article/10.3390/ijms23073681/s1.
