# Supplementary material for: Comparative Transcriptome Analysis between Two Potato Cultivars in Tuber Induction to Reveal Associated Genes with Anthocyanin Accumulation
Source: Int J Mol Sci. 2022 Mar 27;23(7):3681. doi: 10.3390/ijms23073681 (PMC8998591; doi:10.3390/ijms23073681)
Supplement: Supplementary file 1 [file ijms-23-03681-s001.zip › Supplemental_figures.pdf]

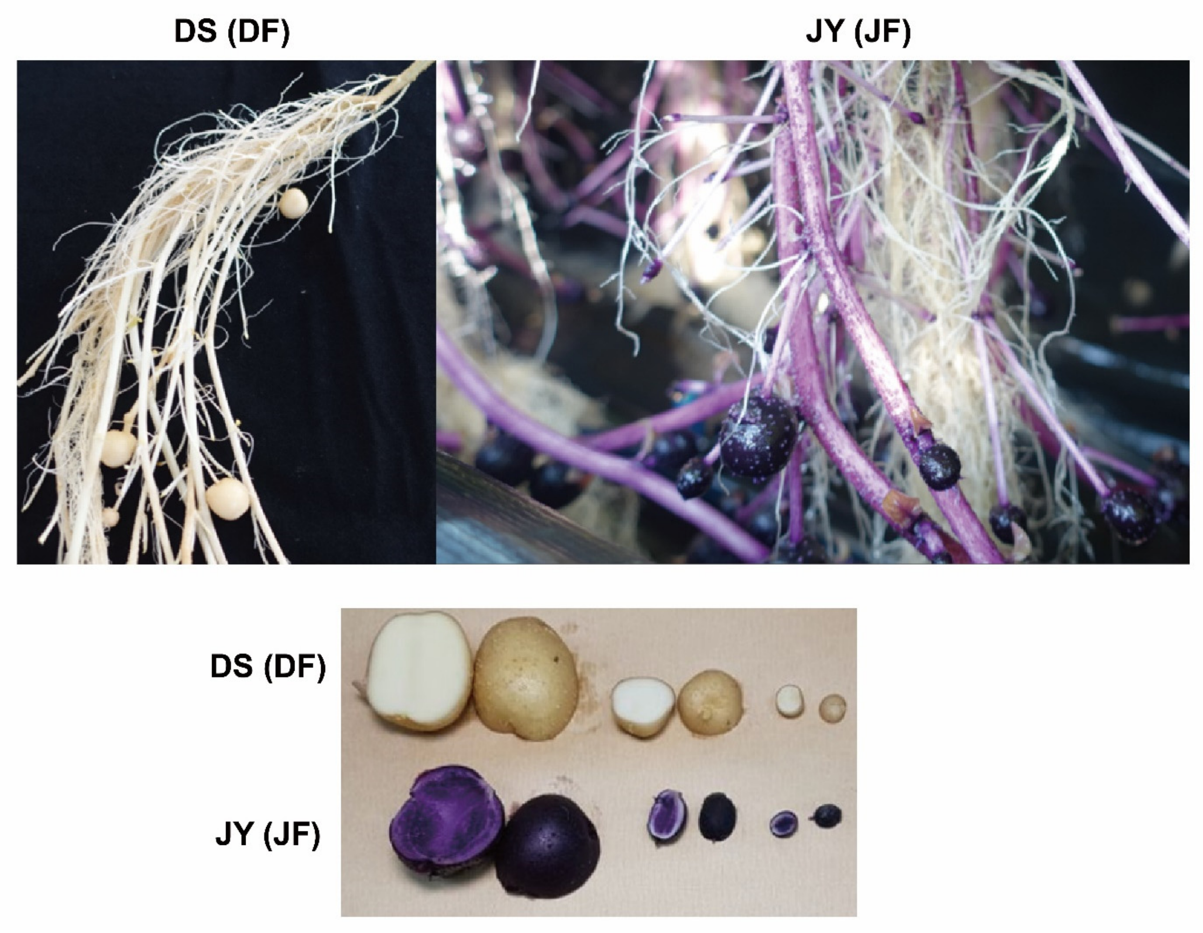

Figure S1. Subterranean roots and stolons of nutrient culture of AT and JY. Upper panels showed potato roots bearing tubers. Bottom photo showed cross-sectioned tubers with different sizes. Skin and flesh tissues from JY(NC) tubers (4-10 mm in diameter) were used in RNA_seq and expression study. DS, Daeseo; JY, Jayoung; NC, nutrient culture; DF, Flesh tissue of Daeseo; JF, Flesh tissue of Jayoung

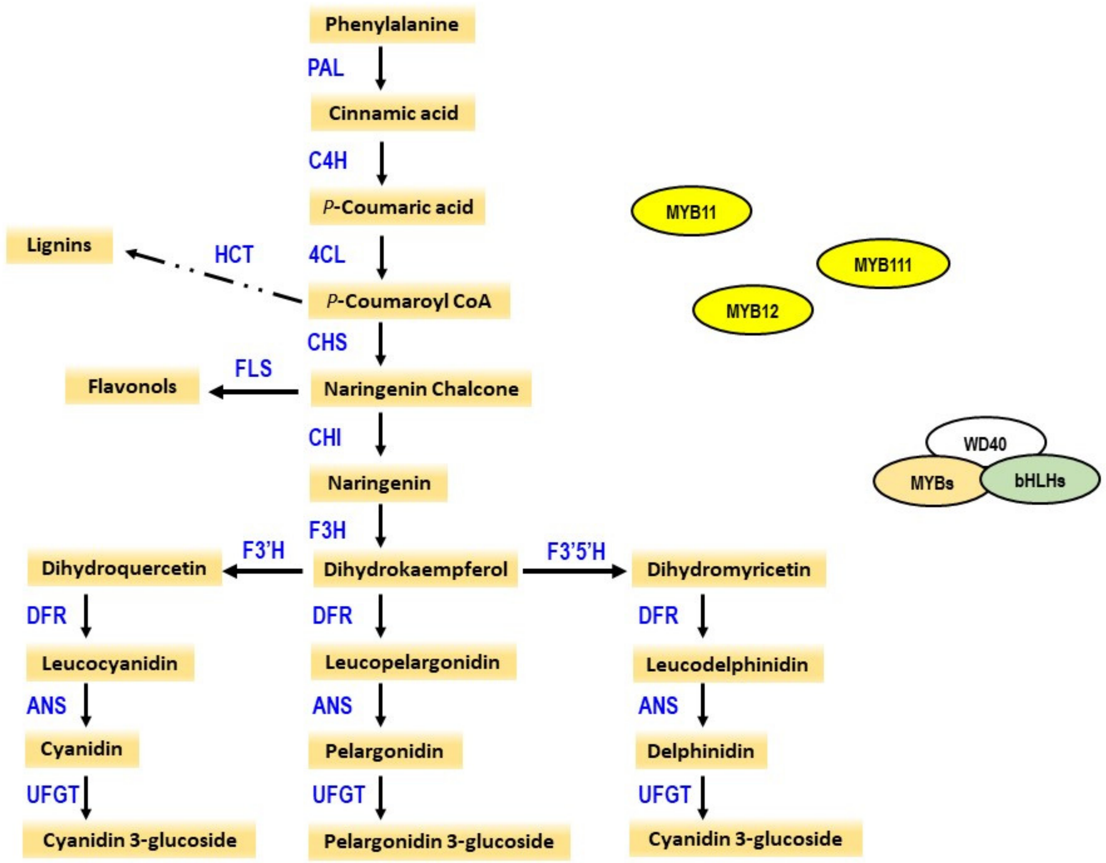

Figure S2. Graphical representation of anthocyanin biosynthesis in planta. Each squares indicates ligand molecules. Blue letters indicate enzymes catalyzes each steps. Circles indicate well-known transcription factors regulates anthocyanin biosynthesis.

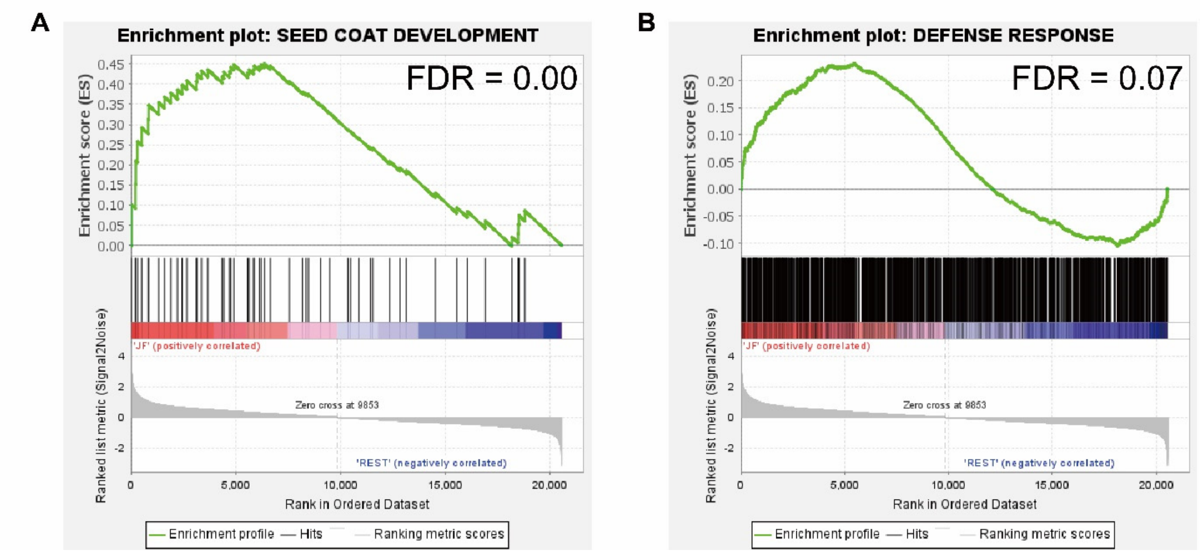

Figure S3. GSEA results of common DEG on other GO terms indicate JF is also positively enriched with seed coat development. **(A**) GSEA result for seed coat development genes. **(B)** GSEA result for defense response genes. GSEA query and method was same with Figure 4.

A

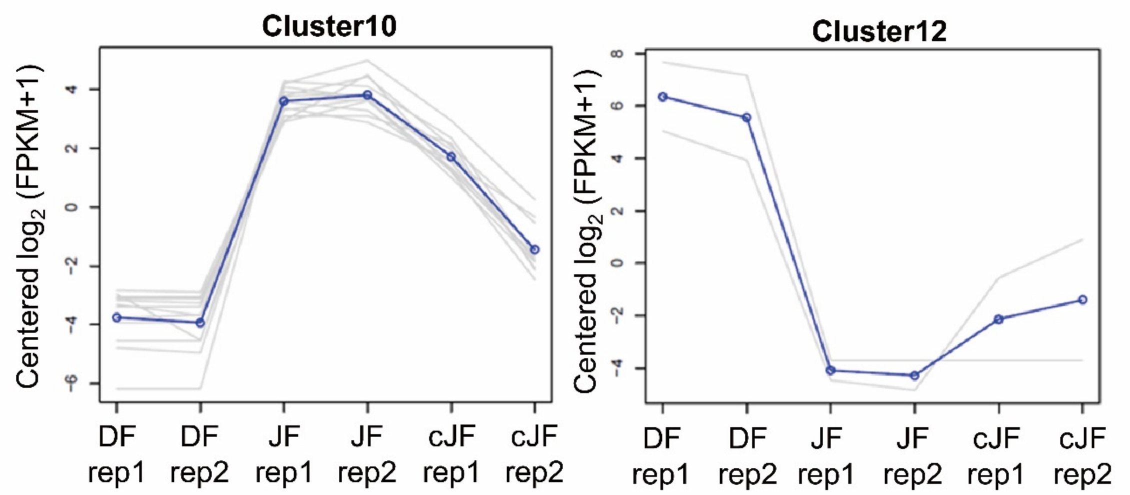

B

	Potato ID	AGI	TAIR description	Ensembl description
Cluster10	PGSC0003DMT400058554	AT4G22880	AT4G22880 protein	Anthocyanin synthase
	PGSC0003DMT400011627	AT4G15550	UDP-glycosyltransferase 75D1	Anthocyanin 5-O-glucosyltransferase
	PGSC0003DMT400009287	AT5G42800	Dihydroflavonol reductase	Dihydroflavonol 4-reductase
	PGSC0003DMT400041528	AT3G26040	HXXXD-type acyl-transferase family protein	Anthocyanin acyltransferase
	PGSC0003DMT400016512	AT1G67980	Putative caffeoyl-CoA O-methyltransferase At1g67980	Caffeoyl-CoA O-methyltransferase
	PGSC0003DMT400036281	AT4G09460	Transcription repressor MYB6	Transcription factor R2R3-MYB
	PGSC0003DMT400001124	AT5G07990	TT7	Flavonoid 3',5'-hydroxylase
	PGSC0003DMT400055458	AT5G48810	Cytochrome B5 isoform D	Cytochrome B5
	PGSC0003DMT400081478	AT1G51200	Zinc finger A20 and AN1 domain-containing stress-associated protein 2	Stress-associated protein 3
	PGSC0003DMT400026355	AT4G25640	Protein DETOXIFICATION	Anthocyanin permease
	PGSC0003DMT400030107	AT1G24430	F21J9.9	Anthocyanin acyltransferase
	PGSC0003DMT400043105	AT3G03190	Glutathione S-transferase F11	Glutathione S-transferase
	PGSC0003DMT400049165	AT5G13930	Chalcone synthase family protein	Chalcone synthase 2
Cluster12	PGSC0003DMT400076178	AT5G13930	Chalcone synthase family protein	CHS1B
	PGSC0003DMT400015767	-	-	Metallocarboxypeptidase inhibitor

Figure S4. Cluster analysis in DF, JF and cFJ indicates most of genes correlates with anthocyanin phenotype were flavonoid biosynthetic genes. **(A)** Cluster10 and cluster12 were JF specifically up-regulated or down-regulated genes. **(B)** Cluster10 and Cluster12 were enriched with anthocyanin related genes. Pink shaded rows are anthocyanin related genes.

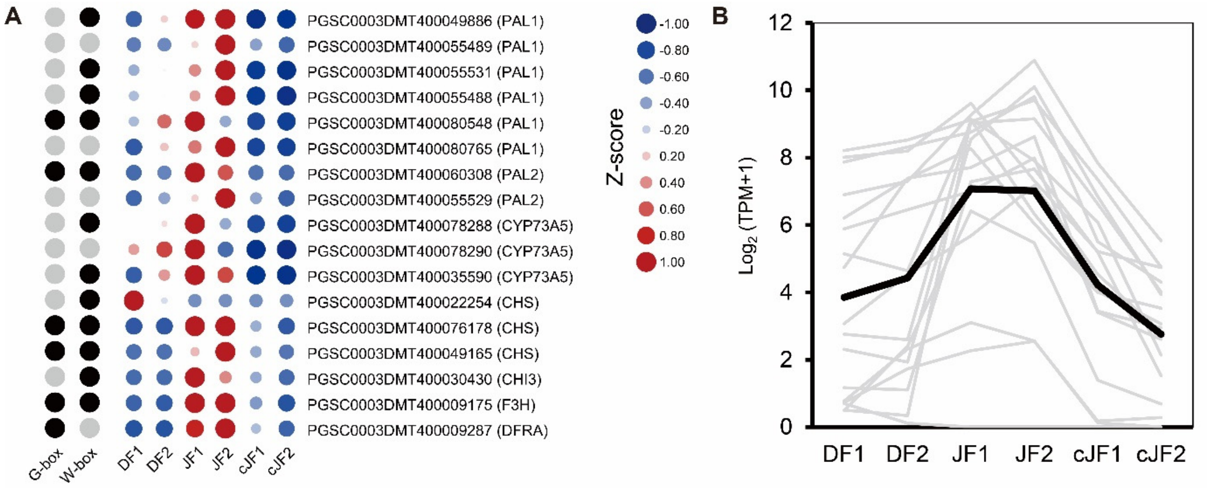

Figure S5. Expression patterns of putative target genes of WRKY44 and TT8 suggested WRKY44 and TT8 being putative regulator of JF specific anthocyanin accumulation. **(A)** Expression pattern and motif analysis of flavonoid biosynthetic pathway genes. Black circle indicates either motif exists in 1 kb promoter region while pale grey circle indicates non-exist. **(B)** Expression patterns of flavonoid biosynthetic genes shown in A. Grey lines indicate expression levels of each genes while black line indicates average gene expression patterns.

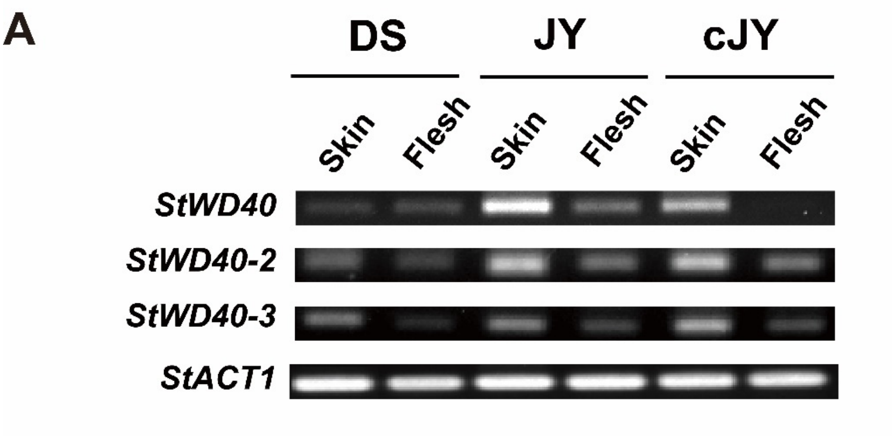

Fw primer	Fw sequence	Rv primer	Rv sequence	amplicon size
StWD40-F	5'-AGAGATCAACCTAGTCGCAAGCG	StWD40-R	5'-TGTGTATCAGCATATGCTGGAGCA	250
StWD40-2F	5'-GATGACGCGATCAACACACT	StWD40-2R	5'-CCTATCACAAGCACCCGAAT	210
StWD40-3F	5'-GTTCATGCTCTGGCTCTTCC	StWD40-3R	5'-GCAGGTCTCCTCCATTTTGA	226

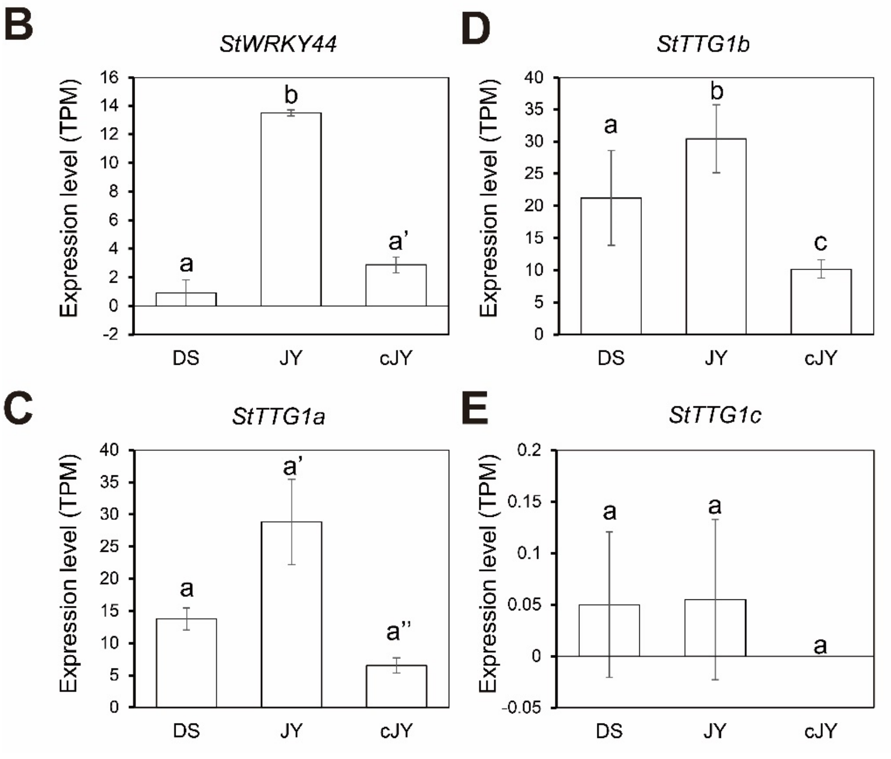

Figure S6. Expression patterns of *StWD40*, *StWRKY44* and *StTTG1* homologs correlates with anthocyanin accumulation phenotype. **(A)** RT-PCR of *StWD40* homolog genes. Used primer set is listed below gel image. **(B-E)** Expression patterns of *StWRKY44* and *StTTG1* homologs from RNA-seq data. Expression levels of each genes were shown by TMM-normalized TPM values. Letters represent significant differences based on one-way ANOVA and Tukey's test ($P < 0.05$). **(B)** Relative expression level of *StWRKY44*. **(C)** Relative expression level of *StTTG1a*. **(D)** Relative expression level of *StTTG1b*. **(E)** Relative expression of *StTTG1c*.

A

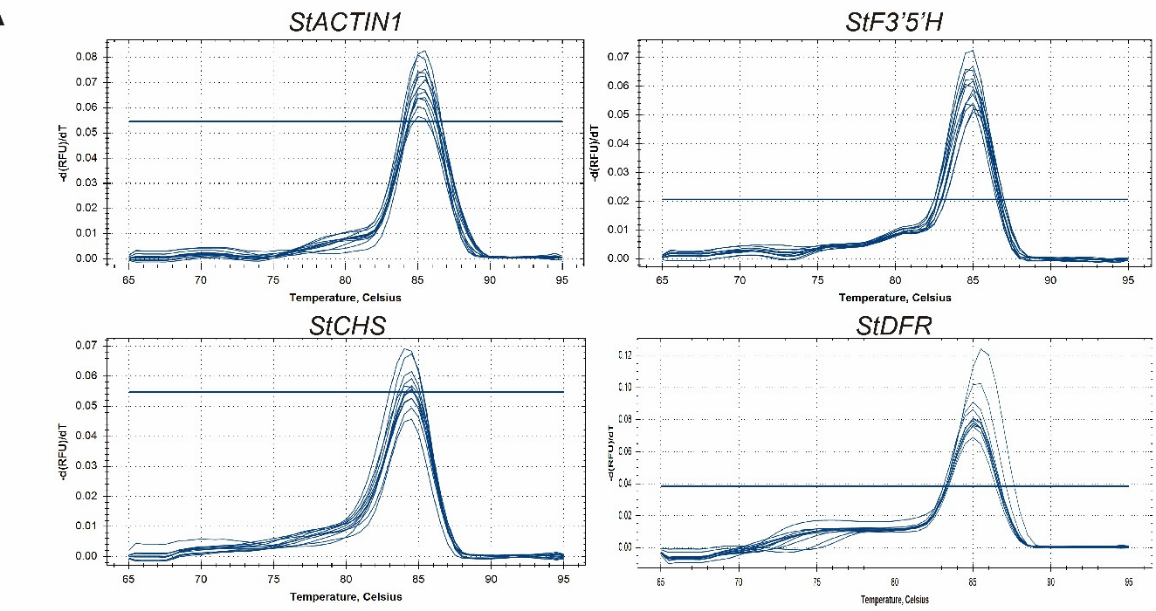

B

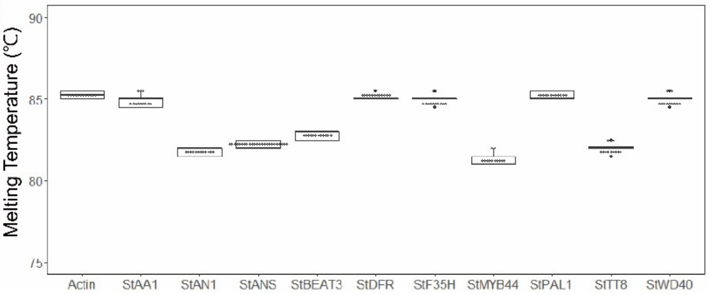

C

Sample		Concentration (ng/ul)	260/280	used volume (ul)	real RNA amount (ng)
DS	Skin1	499.88	2.132	6	2999.28
	Skin2	553.36	2.125	5.4	2988.144
	Flesh1	1159.4	2.146	2.6	3014.44
	Flesh2	1107.1	2.077	2.7	2989.17
JY	Skin1	863.2	2.225	3.5	3021.2
	Skin2	499.76	2.143	6	2998.56
	Flesh1	1572.8	2.156	1.9	2988.32
	Flesh2	826.6	2.211	3.6	2975.76
cJY	Skin1	242.84	2.096	12.4	3011.216
	Skin2	288	2.053	10.4	2995.2
	Flesh1	991.44	2.21	3	2974.32
	Flesh2	435.76	2.135	6.9	3006.744

Figure S7. Detailed preliminary data on qRT-PCR ensures integrity of qRT-PCR data. **(A)** Melting curve of genes used in Figure 5. **(B)** Melting temperature data in box plot with dots. Error bar indicates standard deviation. Some of the primers were not shown in main figure. **(C)** RNA quantification data used in cDNA library preparation for qRT-PCR in table. Purity was checked with 260/280.
