# Supplementary figures and images for: Comparative Transcriptome Analysis between Two Potato Cultivars in Tuber Induction to Reveal Associated Genes with Anthocyanin Accumulation
Source: Int J Mol Sci. 2022 Mar 27;23(7):3681. doi: 10.3390/ijms23073681 (PMC8998591; doi:10.3390/ijms23073681)

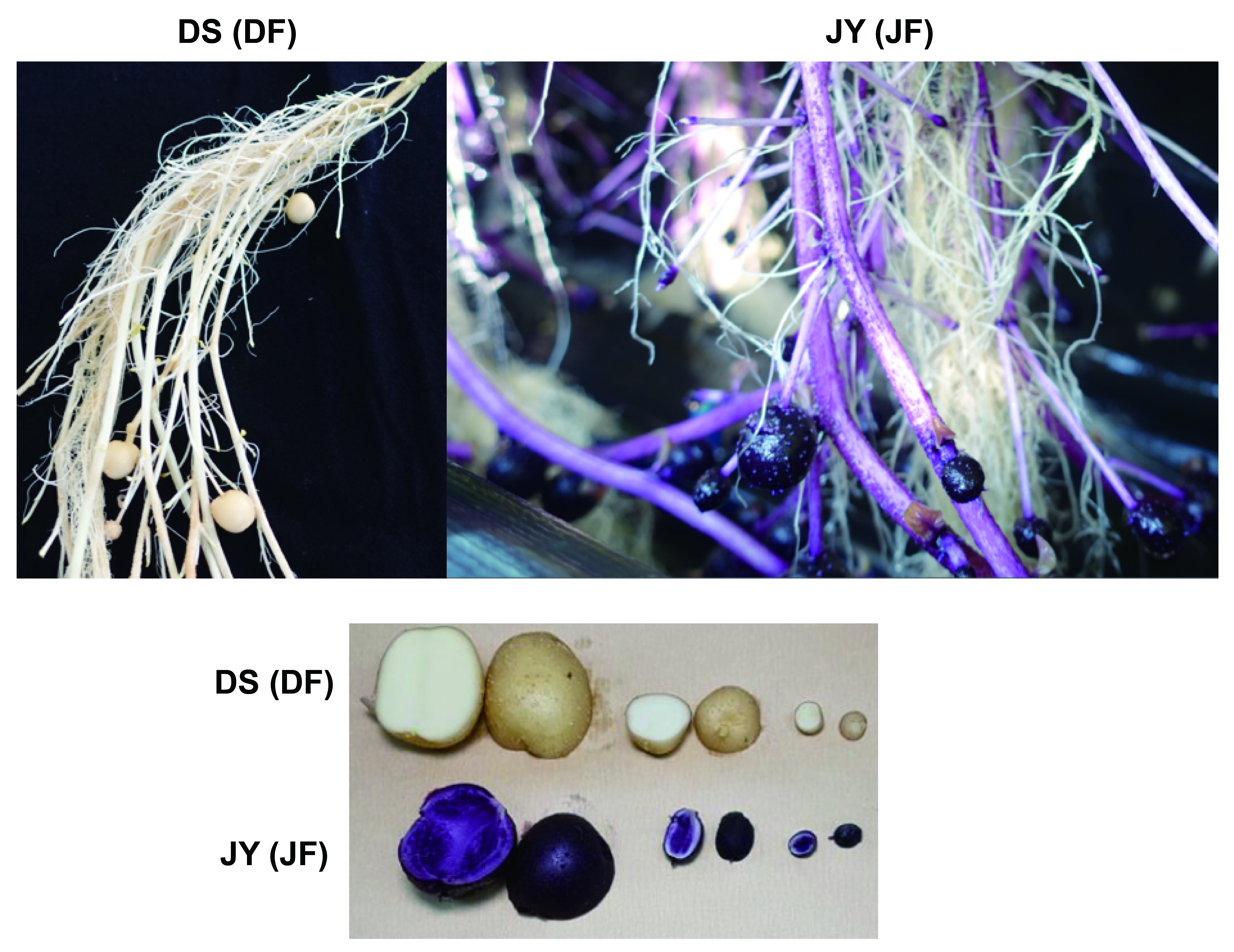

Supplement: Supplementary file 1 [file ijms-23-03681-s001.zip › FigureS1.jpg]

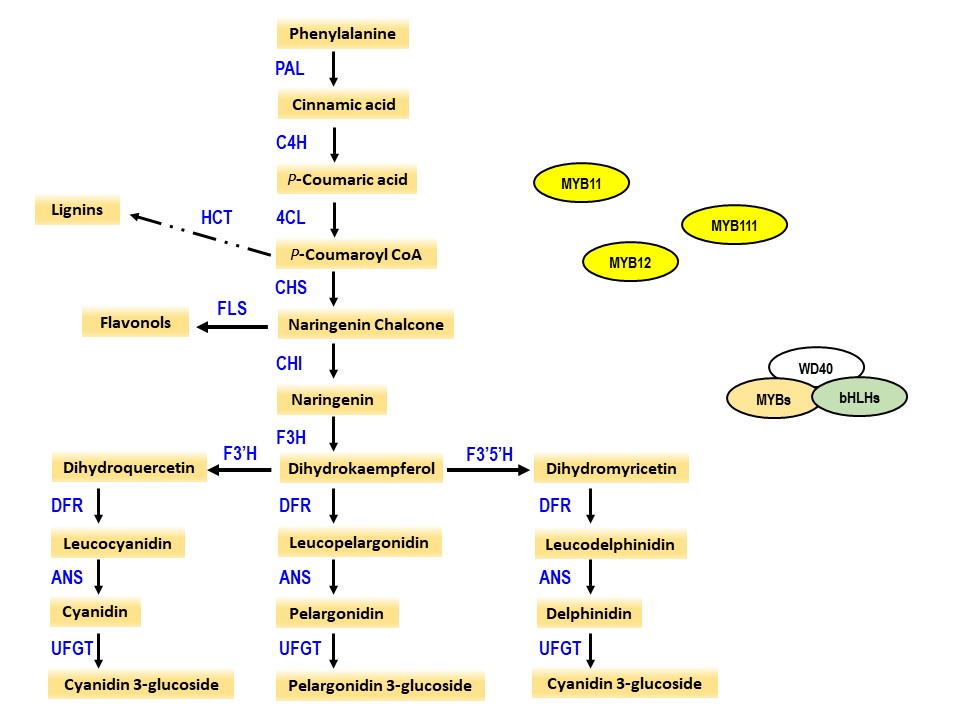

Supplement: Supplementary file 1 [file ijms-23-03681-s001.zip › FigureS2.jpg]

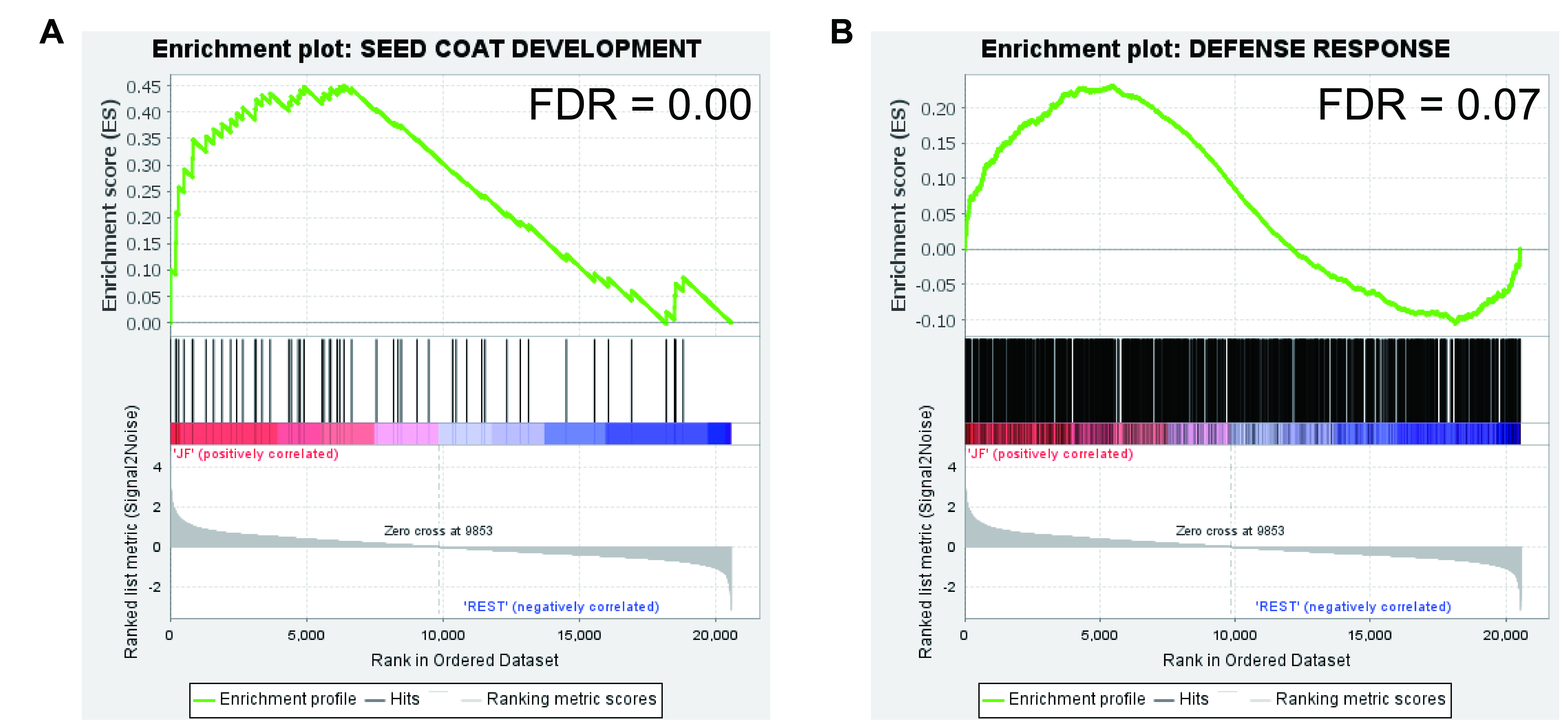

Supplement: Supplementary file 1 [file ijms-23-03681-s001.zip › FigureS3.jpg]

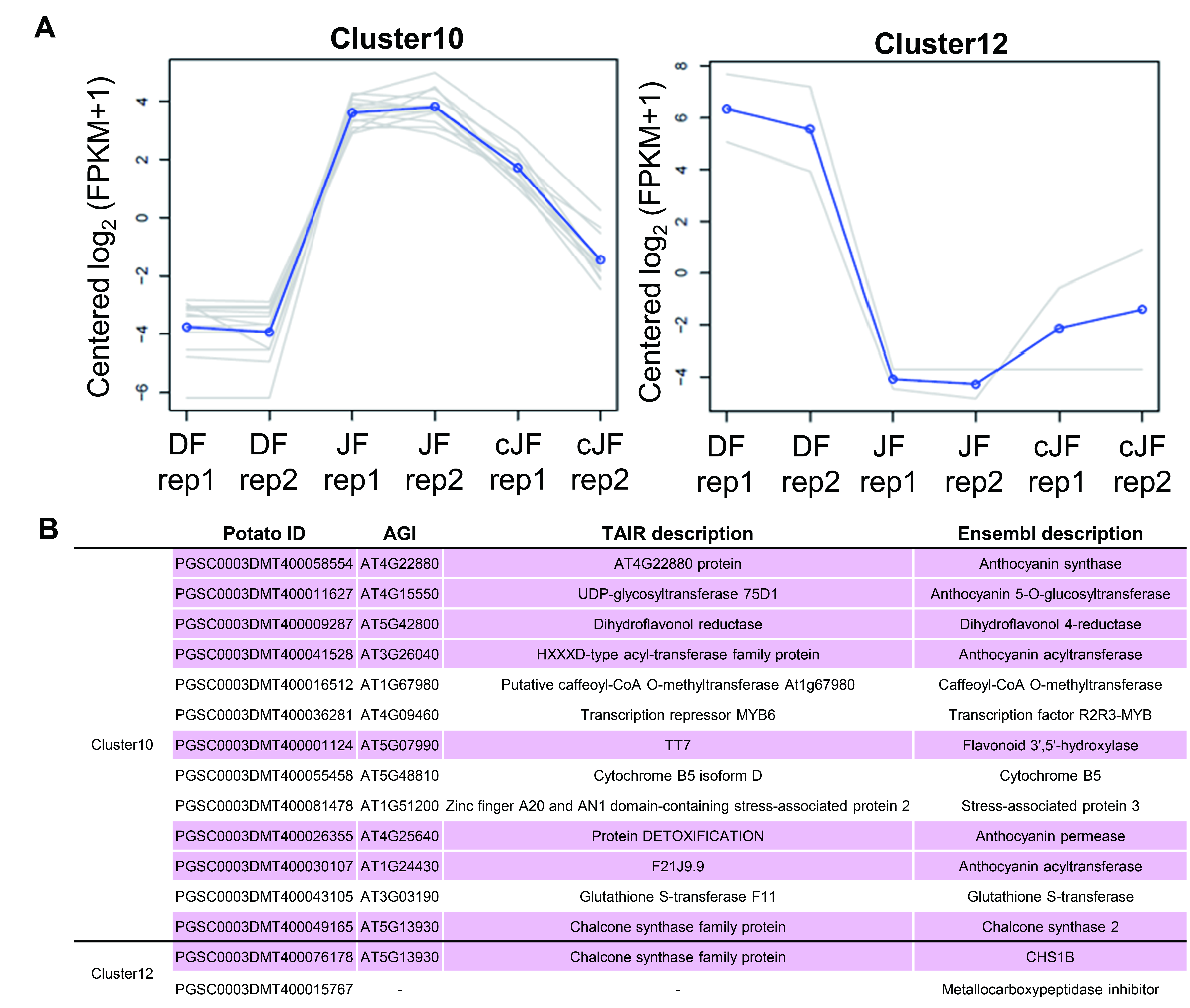

Supplement: Supplementary file 1 [file ijms-23-03681-s001.zip › FigureS4.jpg]

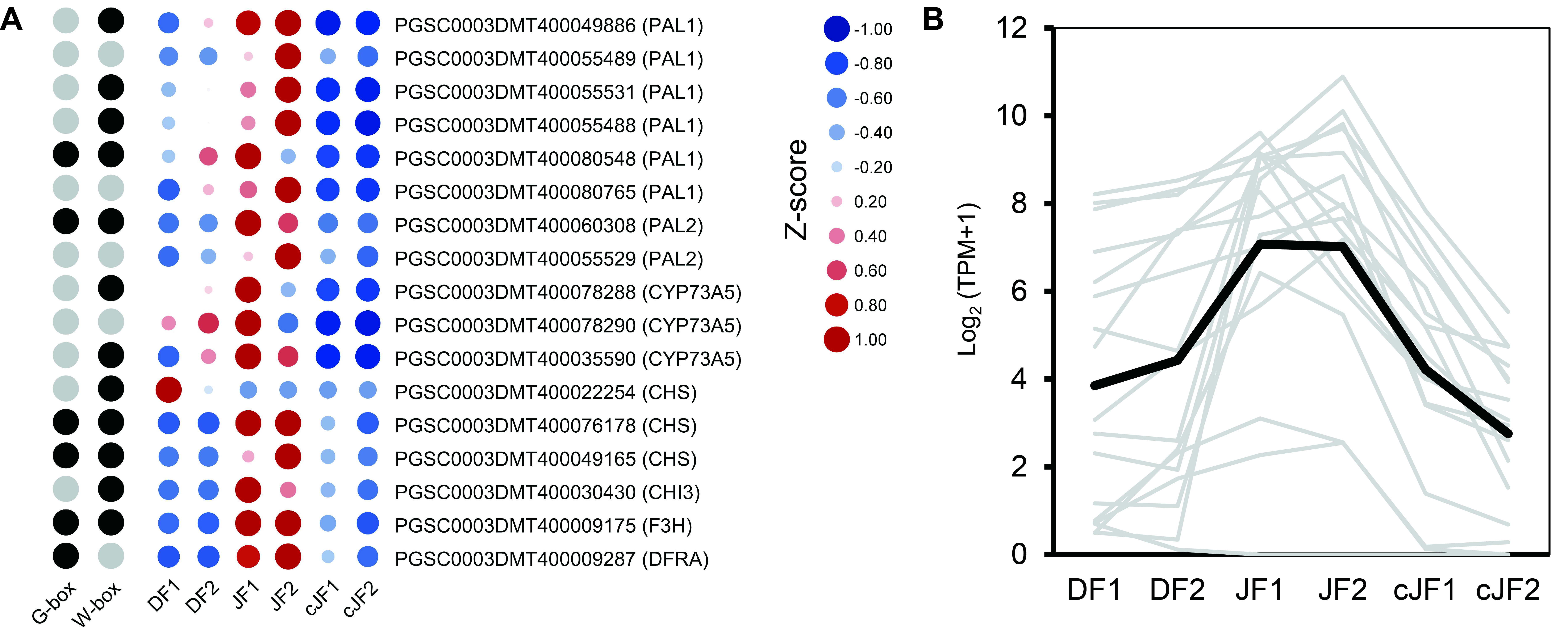

Supplement: Supplementary file 1 [file ijms-23-03681-s001.zip › FigureS5.jpg]

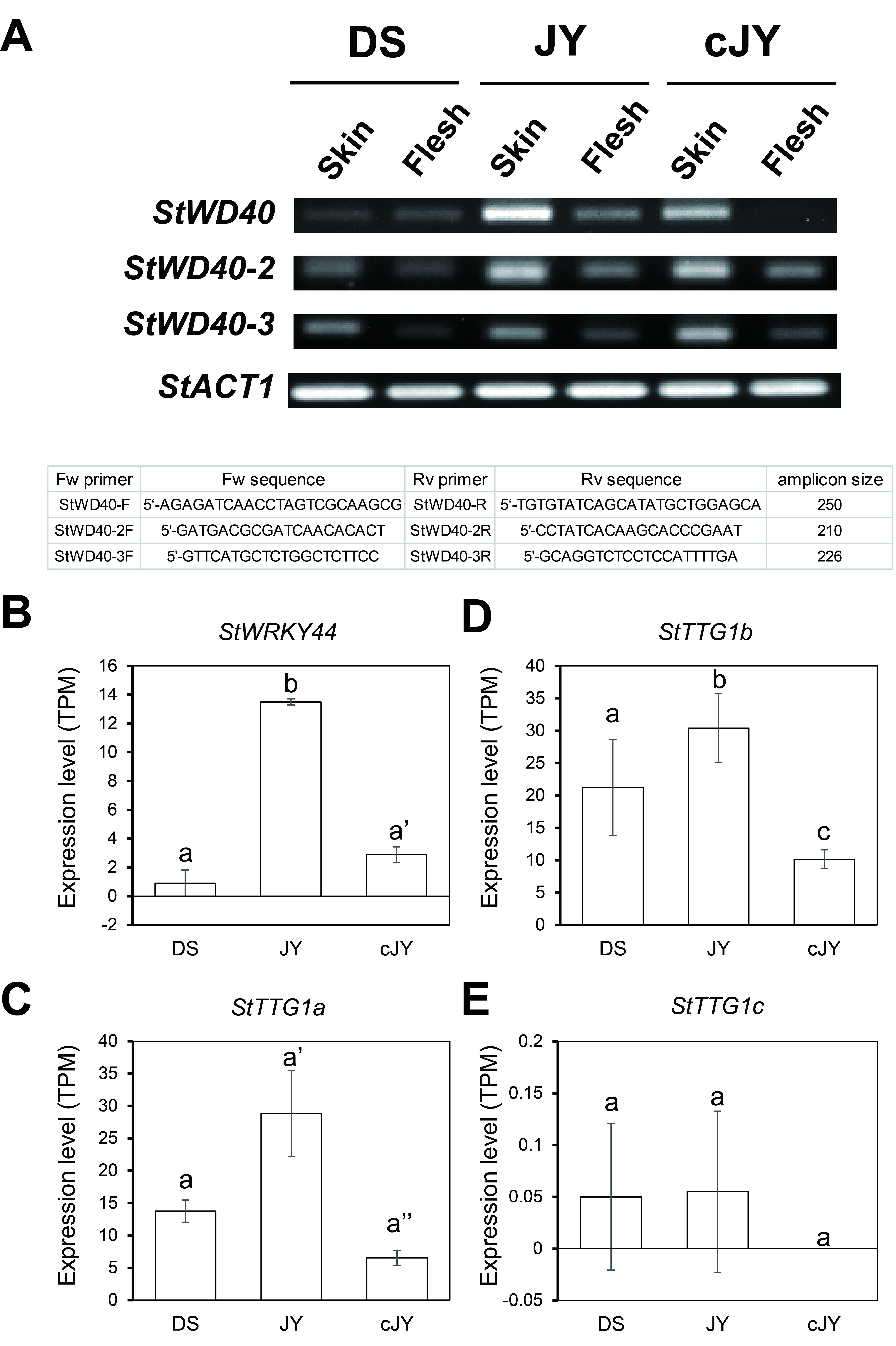

Supplement: Supplementary file 1 [file ijms-23-03681-s001.zip › FigureS6.jpg]

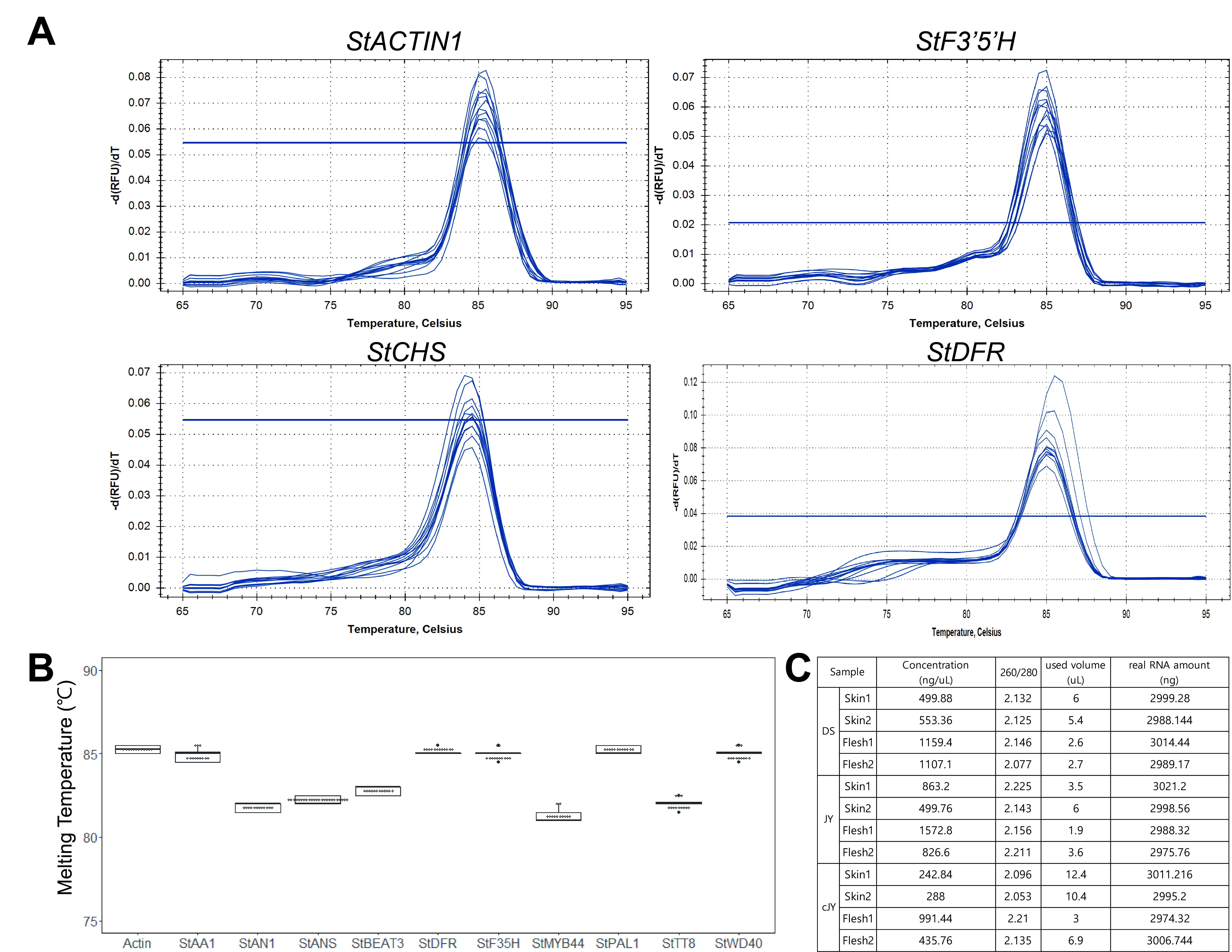

Supplement: Supplementary file 1 [file ijms-23-03681-s001.zip › FigureS7.jpg]

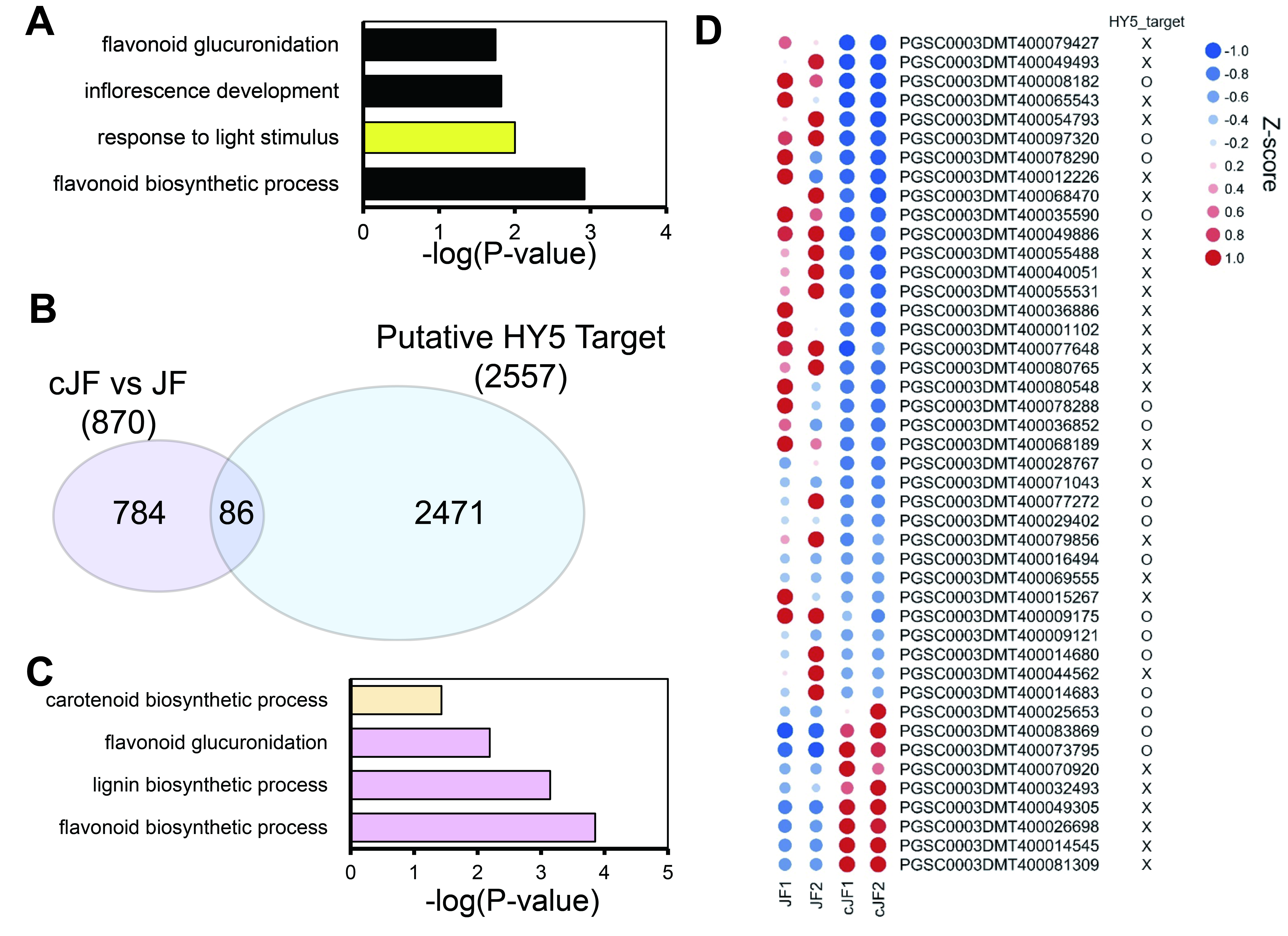

Supplement: Supplementary file 1 [file ijms-23-03681-s001.zip › FigureSS1.jpg]
